# Genome-wide identification and analysis of MAPK and MAPKK gene family in Chinese jujube (*Ziziphus jujuba* Mill.)

**DOI:** 10.1186/s12864-017-4259-4

**Published:** 2017-11-09

**Authors:** Zhiguo Liu, Liman Zhang, Chaoling Xue, Hu Fang, Jin Zhao, Mengjun Liu

**Affiliations:** 1 Research Center of Chinese Jujube, Hebei Agricultural University, Baoding, China; 2 College of Life Science, Hebei Agricultural University, Baoding, China; 3 BGI-Shenzhen, Shenzhen, China

**Keywords:** Chinese jujube, MAPKs, MAPKKs, Bioinformatics analysis, Gene expression

## Abstract

**Background:**

Chinese jujube (*Ziziphus jujuba* Mill.) is one of the most important members in the Rhamnaceae family. The whole genome sequence and more than 30,000 proteins of Chinese jujube have been obtained in 2014. Mitogen-activated protein kinase cascades are universal signal transduction modules in plants, which is rapidly activated under various biotic and abiotic stresses. To date, there has been no comprehensive analysis of the MAPK and MAPKK gene family in Chinese jujube at the whole genome level.

**Results:**

By performing a series of bioinformatics analysis, ten MAPK and five MAPKK genes were identified from the genome database of Chinese jujube, and then compared with the homologous genes from *Arabidopsis*. Phylogenetic analysis showed that ZjMAPKs was classified into four known groups, including A, B, C and D. ZjMAPKs contains five members of the TEY phosphorylation site and five members with the TDY motif. The ZjMAPKK family was subsequently divided into three groups, A, B and D. The gene structure, conserved motifs, functional annotation and chromosome distribution of ZjMAPKs and ZjMAPKKs were also predicted. ZjMAPKs and ZjMAPKKs were distributed on nine pseudo-chromosomes of Chinese jujube. Subsequently, expression analysis of ZjMAPK and ZjMAPKK genes using reverse transcription PCR and quantitative real-time PCR was carried out. The majority of ZjMAPK and ZjMAPKK genes were expressed in all tested organs/tissues with considerable differences in transcript levels indicating that they might be constitutively expressed. Moreover, *ZjMKK5* was specific expressed in early development stage of jujube flower bud, indicating it plays some roles in reproductive organs development. The transcript expression of most ZjMAPK and ZjMAPKK genes was down-regulated in response to plant growth regulators, darkness treatment and phytoplasma infection.

**Conclusions:**

We identified ten ZjMAPK and five ZjMAPKK genes from the genome database of Chinese jujube, the research results shown that *ZjMPK*s and *ZjMKK*s have the different expression patterns, indicating that they might play different roles in response to various treatments. The results provide valuable information for the further elucidation of physiological functions and biological roles of jujube MAPKs and MAPKKs.

**Electronic supplementary material:**

The online version of this article (10.1186/s12864-017-4259-4) contains supplementary material, which is available to authorized users.

## Background

Mitogen-activated protein kinases (MAPK) and mitogen-activated protein kinases kinases (MAPKK) are significant families of serine/threonine kinases, which have been found to involve in wide variety of metabolism pathway include cell division, developmental processes and defense response [1–5]. MAPK cascades comprise three kinds of kinases: MAPK kinase kinase (MAPKKK/MEKK), MAPK kinase (MAPKK/MEK) and MAPK (MPK) [6], and they are gradually phosphorylated by upstream signal stimuli. MAPK substrates play important roles in regulating various intracellular reactions and responding to extra stimulation.

To date, the most extensively studied plant MAPK cascades genes are in *Arabidopsis*. After the completion of the *Arabidopsis* whole genome sequencing, 80 MAPKKKs, 10 MAPKKs and 20 MAPKs were screened firstly at genome level [7, 8]. Then, the members of MAPK cascades from rice, maize, *Brachypodium distachyon*, tobacco, *Brassica rapa*, *Gossypium raimondii*, poplar*,* apple, grape and mulberry at whole genome level were successively identified as well [9–18]. More bioinformatics and function studies of MAPK cascades genes were further carried out in these plants. Plant MAPKs (MPKs) can be divided into four groups (Group A, B, C and D) based on sequence alignment and the phylogenetic analysis, which have two phosphorylation motifs of TEY and TDY. Groups A, B and C of MAPKs share the TEY motif, while group D have the TDY motif and a long C terminal sequence [7]. MAPKKs (MKKs) were also composed of four groups (Group A, B, C and D) in *Arabidopsis thaliana* and rice [19]. Although the structure of plant MAPK cascades genes is highly conserved, they have been proved to involve in various biotic and abiotic stresses, hormones, cell division, plant growth and developmental processes. For example, in *Arabidopsis*, *AtMPK3* and *AtMPK6* have been demonstrated to be the significant regulators in response to pathogen infections and abiotic stresses [20], while *AtMPK9* and *AtMPK12* were highly expressed in guard cells and regulated ROS-mediated ABA signaling positively [21]. In addition, overexpressing *GhMPK16* in *Arabidopsis* has been indicated that it have significant resistance to bacterial pathogens, fungi, drought and H_2_O_2_ [22].

Chinese jujube (*Ziziphus jujuba* Mill.) has experienced more than 7000 years in artificial cultivation, which is one of the most important member in the Rhamnaceae family [23]. Its fruit is rich in vitamin C and sugar [24]. Moreover, the jujube trees have stronger adaption to various biotic and abiotic stresses, especially drought and salinity. The whole genome sequence and more than 32,000 proteins of Chinese jujube have been obtained recently [25], which provided data resource for genome-wide analyses of specific gene families. To date, there have been no studies into the jujube MAPK and MAPKK gene family at the whole genome level. The information of MAPKs and MAPKKs of Chinese jujube is also very limited in the public databases.

Taking advantage of the available jujube genome database, a genome-wide search for the homologues of the MAPK and MAPKK families in jujube was performed in this study. Five MAPKK and ten MAPK genes were identified from jujube genome. The phylogenetic analysis, gene structure, conserved motifs, functional annotation and chromosome distribution of ZjMAPKs and ZjMAPKKs were also predicted. In addition, expression profile analyses of above genes using the reverse transcription PCR (RT-PCR) and quantitative real-time PCR (qRT-PCR) were performed in different materials.

## Methods

### Plant materials, growth conditions and treatments

Seven different tissue samples (root, bearing shoot, secondary shoot, leaf, flower bud, flower, and fruit) were collected separately from three trees and used for organ-specific expression analysis. The floral organs were collected at 7 different development stages for qRT-PCR expression analysis, as it shown in Additional file 1: Figure S1.

The cultivar of plantlet which used for plant hormone and darkness experiment was *Ziziphus jujuba* Mill. ‘Dongzao’. All plantlets were collected from Research Centre of Chinese Jujube, Hebei Agricultural University and cultured in a growth cabinet with a photoperiod of 16 h artificial light and 8 h darkness at 26 ± 2 °C.

For α-Naphthalene acetic acid (NAA), indole-3-butyric acid (IBA) and 6-benzyladenine (6-BA) treatments, the plantlets were subjected to 0.5 mg/L IBA, 1 mg/L 6-BA and 1 mg/L NAA, respectively. Plantlets incubated on Murashige and Skoog (MS) medium without plant growth regulators were used as control. All the samples for RNA extractions were collected at 20 days after treatments. For darkness treatment, the plantlets were cultured in a dark chamber and collected at 0 d, 4 d, 8 d, 12 d and 16 d after darkness treatment for RNA isolation. The samples with 0 d darkness treatment were used as control.

For the biotic stress treatment, ‘Dongzao’ plantlets infected with jujube witches’ broom (JWB) were used as test group. JWB, caused by phytoplasma, is the most serious and destructive disease for jujube tree cultivation. At the same time, healthy plantlets of ‘Dongzao’ were used as control group. All plantlets were cultured in MS medium without plant growth regulators.

Three biological replicates were collected in each treatment. The samples were stored at −80 °C for RNA extraction and expression analysis.

### Cloning of the *ZjMPK4* gene

*ZjMPK4* partial gene sequence was identified from Chinese jujube genome data. So, homology-based cloning method was used to isolate *ZjMPK4* coding sequence (CDS) from Chinese jujube. According to the gene sequences of *Prunus persica* and *Prunus mume*, Primer Premier 5.0 software was used to design specific primers. The *ZjMPK4* gene-specific primers as follow: *ZjMPK4*-S: 5′- ATGGCTGCCAAGGAGTCAAG-3′, *ZjMPK4*-A: 5′- CTAATGGATTGGGGGTCTGG-3′.

### Identification of ZjMAPKs and ZjMAPKKs in Chinese jujube

To identify potential members of the MAPK and MAPKK gene family in Chinese jujube, the completed jujube genome sequence from the DDBJ/EMBL/GenBank (accession JREP00000000) [25] was used. MAPKs and MAPKKs genes of *Arabidopsis* (All sequences were shown in Additional file 2) were used as queries to search in the whole jujube genome database firstly. After searching for MAPK and MAPKK gene family, the Pfam (http://xfam.org/) and SMART (http://smart.embl-heidelberg.de/) [26] databases were used to confirm that the predicted jujube MAPK and MAPKK proteins. Finally, those genes containing full open reading frame (ORF) were manually analyzed using InterProScan and ClustalX program to confirm conserved domains or motifs of MAPK and MAPKK.

### Gene structure and protein structure analysis of ZjMAPKs and ZjMAPKKs

National Center for Biotechnology Information (NCBI, http://www.ncbi.nlm.nih.gov) ORF finder was used to find putative open reading frames and functional domains were determined by blastp of NCBI as well. The gene structures of the ZjMAPKs and ZjMAPKKs were generated with the GSDS (Gene Structure Display Server: http://gsds.cbi.pku.edu.cn/). Then, the exon-intron structures for individual jujube ZjMAPK and ZjMAPKK genes were checked and measured by aligning the cDNA sequences to their corresponding genomic DNA sequences [27]. The isoelectric points and molecular weights of ZjMAPKs and ZjMAPKKs were obtained with the help of proteomics and sequence analysis tools on the ExPASy Proteomics Server (http://expasy.org/). All of the relevant genes identified in the jujube genomes were aligned using ClustalX program (version 2.0).

### The chromosomal location of ZjMAPKs and ZjMAPKKs

To determine the locations of the two gene families on pseudo-chromosomes, the ZjMAPKs and ZjMAPKKs sequences were further used as query sequences for the BLASTN search against the jujube genome sequence. Each *ZjMAPKs* and *ZjMAPKKs* gene was mapped to the jujube genome according to our previously study [28].

### Multiple sequence alignment and phylogenetic tree construction

The full-length amino acid sequences of the MAPKs (20 AtMAPKs, 10 ZjMAPKs) and MAPKKs (10 AtMAPKKs, 5 ZjMAPKKs) were used for multiple sequence alignment and phylogenetic analyses, respectively. Multiple sequence alignments of the amino acid sequences were conducted with default parameters by using ClustalX program (version 2.0). Phylogenetic analyses based on the protein sequences were performed by the software MEGA5.0 [29]. The unrooted phylogenetic trees were constructed using the neighbor-joining (NJ) method, and the reliability of the trees obtained was tested using bootstrapping with 1000 replicates. Scale bar represents 0.05 amino acid substitution per site.

### Reverse transcription PCR and quantitative real-time PCR analysis

Total RNA was extracted using the TIANGEN RNA Extraction Kit. First-strand cDNA was synthesized using the PrimeScript 1st Strand cDNA Synthesis Kit (Takara, Dalian, China) according to manufacturer’s instructions. The resulting cDNA was then diluted nine-fold and stored at −20 °C for the subsequent RT-PCR and qRT-PCR assays.

For gene expression quantification, specific primers were designed for each ZjMAPKs and ZjMAPKKs gene using the Primer Premier 5.0 software. The information of primers used in this study is listed in the Additional file 3: Table S1. The organ-specific expression analysis was assayed by RT-PCR. The conditions for RT-PCR were as following: 4 min at 94 °C, followed by 30 cycles of 30 s at 94 °C, 30 s at 55–60 °C (varying with specific primers), and 45 s at 72 °C, and a final extension at 72 °C for 7 min to complete the reaction.

The expression of ZjMAPKs and ZjMAPKKs genes in different treatments was examined using qRT-PCR. The qRT-PCR was performed on the Bio-Rad iQ™ 5 using *TransStart* Top Green qPCR SuperMix AQ131 (TransGen Biotech, China). The 25 μL reaction system contains 12.5 μL of 2 × *TransStart* Top Green qPCR SuperMix, 0.5 μL each of 10 μM primers, 1 μL diluted cDNA and 10.5 μL ddH_2_O. The thermal profile was pre-incubation for 30 s at 95 °C, followed by 40 cycles of 5 s at 95 °C, 10 s at 55–60 °C (varying with specific primers), and 10 s at 72 °C. Subsequently, a melting curve analysis was run for one cycle from 55 °C to 90 °C. Three biological replicates were performed for each sample. To normalize the total amount of cDNA present in each reaction, the Chinese jujube *ZjACT* gene was co-amplified as an endogenous control for calibration of relative expression. The relative expression level was calculated by the 2^- △△CT^ method [30].

### Statistical analysis

Data are shown as the means ± SD of independent experiments. Statistical analysis was performed by SPSS 16.0 software using student’s t-test or ANOVA test method. *P* < 0.05 and *P* < 0.01 were considered as significant and highly significant difference, respectively.

## Results

### Identification of ZjMAPKs and ZjMAPKKs in Chinese jujube

**Fig. 1:**
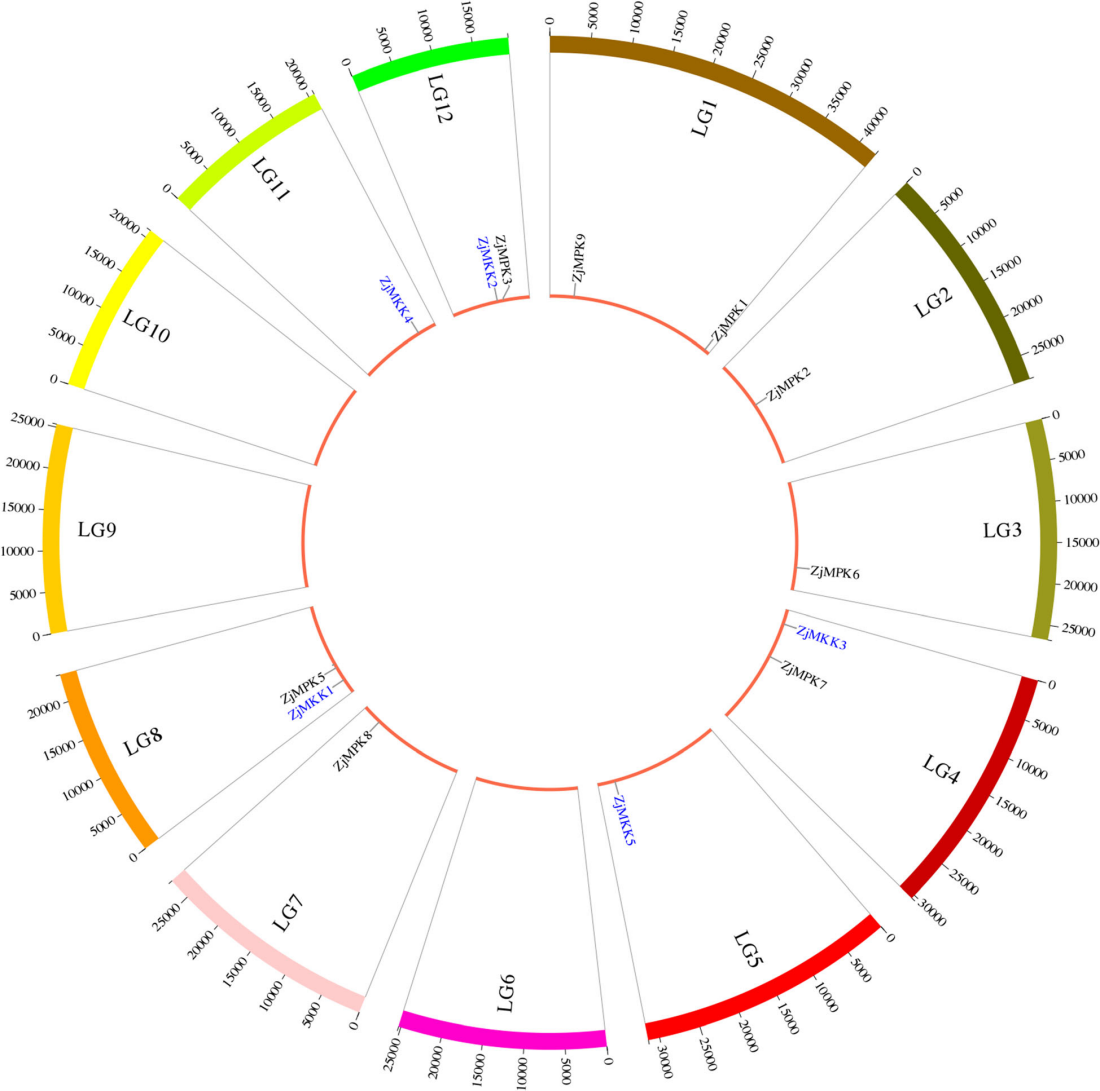
Positions of ZjMPK and ZjMKK genes on the jujube chromosomes. Genes were mapped to the jujube chromosomes via the Circos tool. The jujube chromosomes were arranged in a circle

From jujube genome, nine typical MAPK genes and five typical MAPKK ones (Table 1) were identified and used for further analysis (All sequences were shown in Additional file 2). One MAPK gene (*ZjMPK4*) was isolated by homology cloning (The sequence was shown in Additional file 2). Sequence alignment of amino acid found that the sequence identity of Chinese jujube ZjMPK4 and *Arabidopsis thaliana* AtMPK4 was 82.85%. The CDS length for ZjMAPK genes ranged from 1125 bp (*ZjMPK5*) to 2166 bp (*ZjMPK8*), and they encoded proteins ranging from 374 to 721 amino acids (aa) in length with an average of 515 aa, with a predicted molecular mass range of 42.63–81.15 KDa, and protein pIs ranging from 5.57 (*ZjMPK1*) to 9.10 (*ZjMPK7*) (Table 1). The CDS length for MAPKK genes ranged from 999 bp (*ZjMKK5*) to 1065 bp (*ZjMKK2*), and they encoded proteins ranging from 332 to 354 amino acids (aa) in length with an average of 342 aa, with a predicted molecular mass range of 36.70–39.50 KDa, and protein pIs ranging from 5.53 (*ZjMKK1*) to 6.88 (*ZjMKK5*) (Table 1). ZjMAPKs and ZjMAPKKs were randomly distributed on 9 chromosomes of Chinese jujube (Table 1 and Fig. 1).

**Table 1:** The information of MAPK and MAPKK gene family in Chinese jujube

Gene family	Gene name	Gene model	Chromosomes	Position	ORF (bp)	Size (aa)	MW(KD)	PI	Types	Group
MAPK	*ZjMPK1*	CCG019246.1	Chr1	scaffold501:141,704:145,538:+	1197	398	45.45	5.57	TEY	A
	*ZjMPK2*	CCG014282.1	Chr2	scaffold312:291,457:294,343:-	1128	375	42.96	5.61	TEY	A
	*ZjMPK3*	CCG004076.1	Chr12	scaffold130:145,282:154,186:+	1497	498	57.29	6.13	TEY	B
	*ZjMPK4*				1140	379	43.36	6.36	TEY	B
	*ZjMPK5*	CCG015764.1	Chr8	scaffold364:308,649:310,334:+	1125	374	43.36	7.60	TEY	C
	*ZjMPK6*	CCG007165.2	Chr3	scaffold164:60,108:64,064:-	1728	575	64.79	9.06	TDY	D
	*ZjMPK7*	CCG004734.3	Chr4	scaffold1368:71,249:76,280:-	1851	616	70.24	9.10	TDY	D
	*ZjMPK8*	CCG026115.1	Chr7	scaffold828:71,756:83,030:+	2166	721	81.15	7.82	TDY	D
	*ZjMPK9*	CCG022866.1	Chr1	scaffold663:208,540:214,001:+	1746	581	66.68	7.28	TDY	D
	*ZjMPK10*	CCG016360.1		scaffold389:214,895:246,410:+	1896	631	71.83	6.66	TDY	D
MAPKK	*ZjMKK1*	CCG011783.1	Chr8	scaffold25:290,193:292,650:+	1062	353	39.19	5.53		A
	*ZjMKK2*	CCG011406.1	Chr12	scaffold24:794,781:798,772:+	1065	354	39.50	6.02		A
	*ZjMKK3*	CCG021919.1	Chr4	scaffold612:169,916:171,832:+	1008	335	36.96	5.76		B
	*ZjMKK4*	CCG028907.1	Chr11	scaffold99:188,221:189,240:+	1020	339	37.78	5.96		D
	*ZjMKK5*	CCG005893.1	Chr5	scaffold1471:17,168:18,166:-	999	332	36.70	6.88		D

### Conserved domains and motifs in ZjMAPKs and ZjMAPKKs

**Fig. 2:**
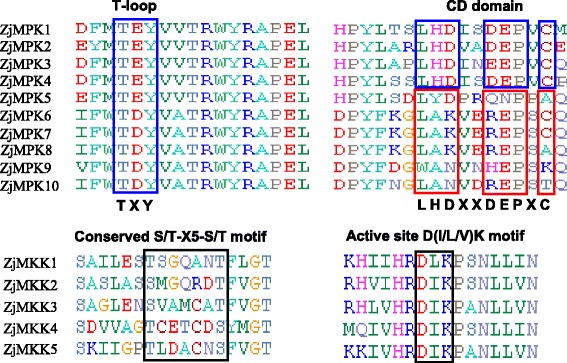
Multiple sequence alignment of the kinase domains of ZjMPK and ZjMKK proteins

Ten ZjMAPK and five ZjMAPKK underwent multiple sequence alignment by ClustalX program (Fig. 2), respectively. The result showed that all the ZjMAPKs and ZjMAPKKs that shared the family joint structure contained conserved protein kinase domains. A TXY motif phosphorylation site existed in ZjMAPKs, among them the TEY type existed in groups A, B and C, whereas group D contained the TDY motif (Fig. 2 and Table 1). Two conserved motifs, S/T-X5-S/T and active site D (I/L/V)K, were found in all ZjMAPKKs (Fig. 2).

### The gene structure of ZjMAPKs and ZjMAPKKs

**Fig. 3:**
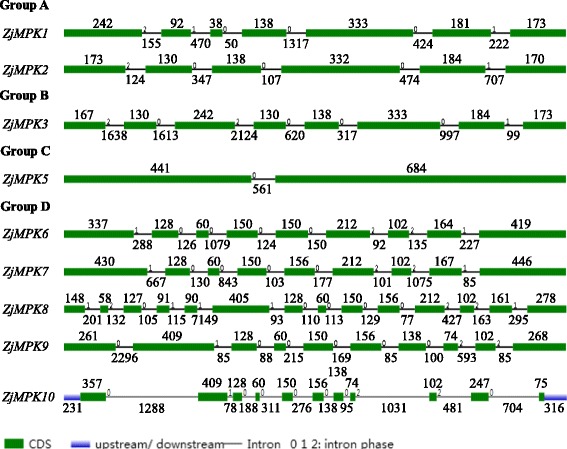
The exon/intron structure of 9 ZjMPK genes in Chinese jujube. Introns and exons are represented by *black lines* and *green boxes* respectively. The length in base pairs of each intron and exon is also indicated

**Fig. 4:**
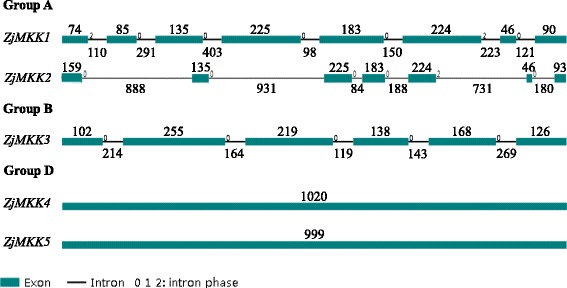
The exon/intron structure of 5 ZjMKK genes in Chinese jujube. Introns and exons are represented by *black lines* and *blue boxes* respectively. Introns and exons are represented by *black lines* and *green boxes* respectively. The length in base pairs of each intron and exon is also indicated. Numbers correspond to the length of the intron and exon

The exon-intron structure diagram for the ZjMAPKs and ZjMAPKKs was also aligned and compared (Figs. 3 and 4). The different groups of ZjMAPKs and ZjMAPKKs have different exon-intron structures, but most members within the same group shared a similar structure and gene length (Figs. 3 and 4). The number introns of ZjMAPKs were from 1 (*ZjMPK5*, one group C gene) to 13 (*ZjMPK8* and *ZjMPK10*, two group D genes) (Fig. 3). In group A, *ZjMPK1* and *ZjMPK2* have seven and six exons, respectively, while all group A MAPKs in *Arabidopsis*, mulberry, poplar and tomato consisted six exons. Group B *ZjMPK3* includes eight exons, which number is more than that of *Arabidopsis*, mulberry, poplar and tomato. Two group C ZjMAPK genes have two exons with strictly conserved sizes, which number are same to the other plant, including *Arabidopsis*, poplar, apple, mulberry and tomato. Moreover, Group C MAPKs of different plants also have similar gene length. The gene structure can provide some useful information of the genomic evolution. The conserved structural patterns of Group C MAPKs in different plants suggested that they may be conserved in evolution. Like other plants, Group D ZjMAPKs possessed a complex distribution of exons and introns, such as both *ZjMPK8* and *ZjMPK10* contained 14 exons.

The number introns of *ZjMAPKKs* were from five to eight (Fig. 4), except the group D *ZjMAPKKs*. Members of group D *ZjMAPKKs* have a completely intron less structure, which is same with that in apple, poplar and *Arabidopsis*. In addition, the number of MAPKK members in all reported plants is always less than that of MAPKs and MAPKKKs, indicating that plant MAPKK genes may be more conserved in evolution than MAPKs and MAPKKKs.

### Multiple sequence alignment and phylogenetic tree construction

In order to evaluate the evolutionary relationship among the MAPK and MAPKK proteins from different species, MAPKs (20 AtMAPKs, 10 ZjMAPKs) and MAPKKs (10 AtMAPKKs, 5 ZjMAPKKs) from Chinese jujube and *Arabidopsis* were subjected to a multiple sequence alignment using the MEGA 5.0 program based on their predicted amino acid sequences. The multiple sequence alignment file was then used for the construction of an unrooted phylogenetic tree. The phylogenetic analysis of the two species indicated that genes that were clustered together might have similar functions.

**Fig. 5:**
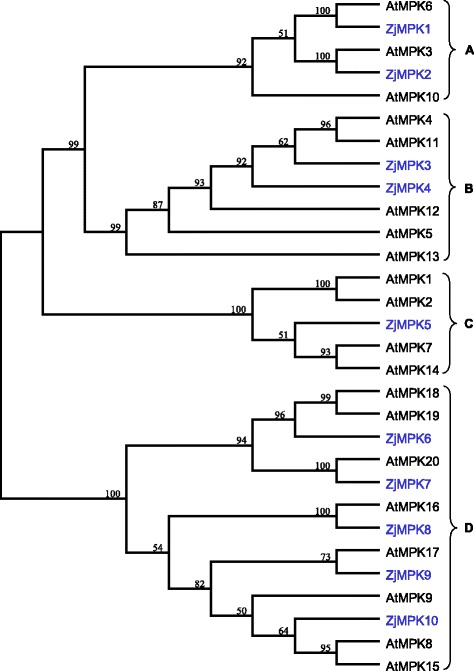
Phylogenetic relationships of MPK genes from Chinese jujube and Arabidopsis

Through phylogenetic analyses of the two species, we found that the ZjMAPKs could also be classified into four groups corresponding to the groups A, B, C and D in *Arabidopsis* (Fig. 5). As shown in Fig. 5, the group D constitutes the largest clade containing 5 ZjMAPKs, followed by groups A (2 genes) and C (2 genes), and the group B include one gene. As shown in Table 2, the group D gene family is the largest subfamily in *Arabidopsis*, maize, rice, *Brachypodium distachyon*, tomato, poplar and apple, too. The size of groups A, B and C is similar in rice, *Brachypodium distachyon*, maize and mulberry, including less genes than in *Arabidopsis*, tomato, poplar and apple, which shows that the amplification of groups A, B and C gene numbers is particularly impressive in the four species.

**Table 2:** The number of the MAPK and MAPKK gene family in Chinese jujube, *Arabidopsis*, Rice, Poplar, Tomato, *Brachypodium distachyon*, Apple, Mulberry and Maize

Gene family	Species	Group A	Group B	Group C	Group D	Total
MAPK	*Arabidopsis*	3	5	4	8	20
	Rice	2	1	2	10	15
	Poplar	4	4	4	9	21
	Tomato	3	4	2	7	16
	*B. distachyon*	2	2	3	9	16
	Apple	5	6	5	10	26
	Mulberry	2	3	2	3	10
	Maize	4	2	2	11	11
	Chinese jujube	2	1	2	5	10
MAPKK	*Arabidopsis*	3	1	2	4	10
	Rice	2	1	2	3	8
	Poplar	3	1	2	5	11
	*B. distachyon*	2	3	2	5	12
	Apple	3	1	2	3	9
	Chinese jujube	2	1	0	2	5

**Fig. 6:**
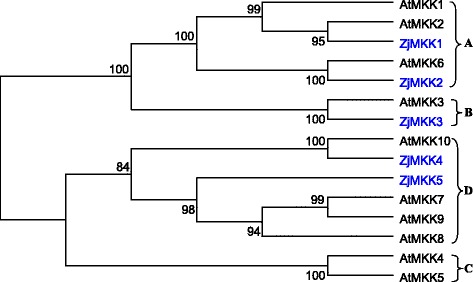
Phylogenetic relationships of MKK genes from Chinese jujube and Arabidopsis

Only 5 MAPKK genes were identified from the Chinese jujube genome, and divided into three subfamilies (groups A, B and D) by the phylogenetic analysis (Fig. 6). ZjMKK1–2, ZjMKK3, and ZjPKK4–5 belong to groups A, B, and D, respectively. In comparison with other plants (*Arabidopsis*, rice, *Brachypodium distachyon*, poplar and apple), it is found that the number of MAPKK gene (from 8 to12) is very conserved and MAPKKs can be divided into four groups (A, B, C, and D) in the other plants (Table 2), but ZjMAPKK genes in group C were not founded.

### Expression patterns of ZjMAPK and ZjMAPKK family genes

**Fig. 7:**
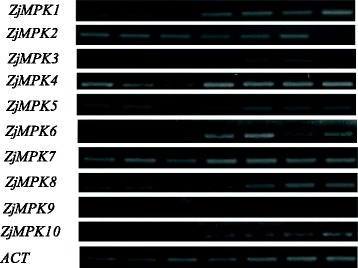
Tissue-specific expression patterns for *ZjMPKs*. *ZjACT* was used as an internal control. From left to right: root, bearing shoot, secondary shoot, leaf, flower bud, flower, and fruit

**Fig. 8:**
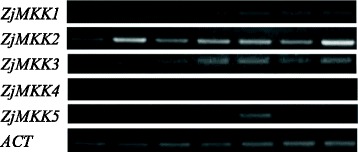
Tissue-specific expression patterns for *ZjMKKs*. *ZjACT* was used as an internal control. From left to right: root, bearing shoot, secondary shoot, leaf, flower bud, flower, and fruit

**Fig. 9:**
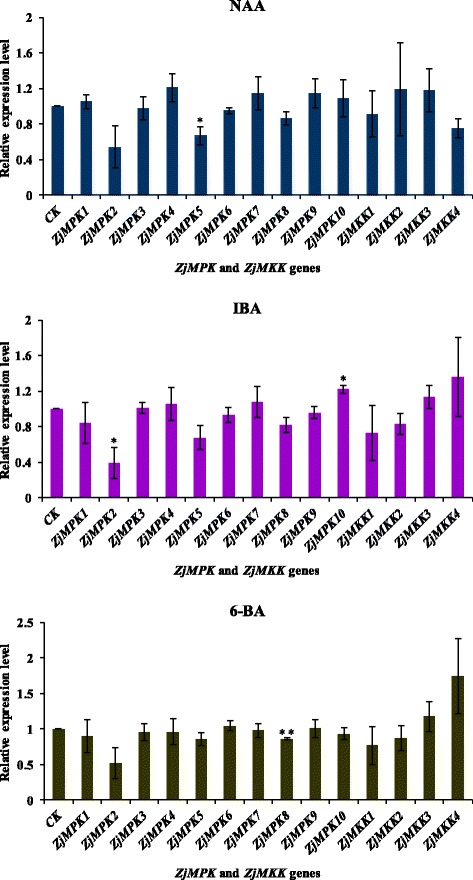
Relative expression of *ZjMPK* and *ZjMKK* genes analyzed by qRT-PCR upon plant growth regulators treatment. *ZjACT* primers were used as the internal standard for each gene. The mean expression value was calculated from 3 independent replicates. The vertical bars indicate the standard deviation. Significant and highly significant difference are shown as * (*P* < 0.05) and ** (*P* < 0.01), respectively

**Fig. 10:**
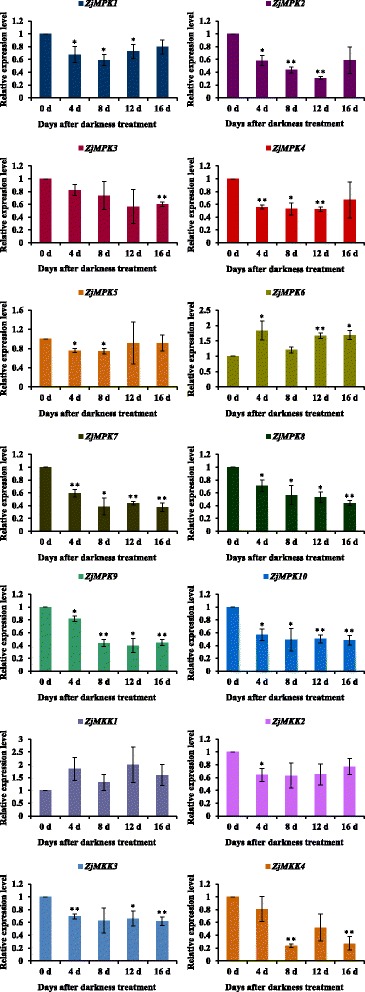
Relative expression of *ZjMPK* and *ZjMKK* genes analyzed by qRT-PCR upon darkness treatment. *ZjACT* primers were used as the internal standard for each gene. The mean expression value was calculated from 3 independent replicates. The vertical bars indicate the standard deviation. Significant and highly significant difference are shown as * (*P* < 0.05) and ** (*P* < 0.01), respectively

**Fig. 11:**
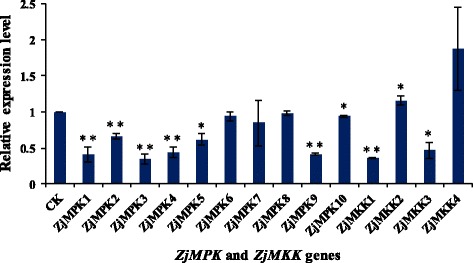
Relative expression of *ZjMPK* and *ZjMKK* genes analyzed by qRT-PCR under phytoplasma infection. *ZjACT* primers were used as the internal standard for each gene. The mean expression value was calculated from 3 independent replicates. The vertical bars indicate the standard deviation. Significant and highly significant difference are shown as * (*P* < 0.05) and ** (*P* < 0.01), respectively

Specific primer sets were designed for each *ZjMAPK* ***s*** and *ZjMAPKKs* (Additional file 3: Table S1) and their expression patterns in different materials were detected by semi-quantitative RT-PCR (Figs. 7, 8) and qRT-PCR (Figs. 9, 10 and 11). Most of the genes were expressed in all tested organs/tissues with considerable differences in transcript levels representing their distinct roles in jujube growth and development. *ZjMKK2* was highly expressed in fruits and *ZjMKK5* was specifically expressed in flower bud (Fig. 8), indicating their functions were involved in reproductive organs development. Moreover, *ZjMKK5* was highly expressed in early stage of floral organ development which was further confirmed by qRT-PCR (Additional file 4: Figure S2). In addition, some genes of the same clade showed similar expression patterns, suggesting possible similar function or functional redundancy.

To study the function of the *ZjMPKs* and *ZjMKKs* in responding to abiotic stresses, the expression of these genes in response to different plant growth regulators and darkness treatment was examined by qRT-PCR. We found that the expression patterns of the two families were different when exposure to various plant growth regulators (Fig. 9). Moreover most members of the two family genes showed similar expressions in response to NAA and IBA treatments except for *ZjMKK4*, this may be due to both NAA and IBA are auxin analogues.

During the darkness treatment, the *ZjMPK* genes exhibited differential expression patterns (Fig. 10). The expression of *ZjMPK7*, *ZjMPK8*, *ZjMPK9* and *ZjMPK10* was decreased significantly at the early stage, and then remained at the low level at the following stage in response to darkness treatment. However, *ZjMPK1*, *ZjMPK2*, and *ZjMPK4* expression were significant decrease from 4 d to 12 d during the darkness treatment, while increased at last stage. *ZjMPK5* expression decreased sharply at first two stages, but there have no obvious changes in followed stage. The expression of *ZjMPK3* was decreased gradually in response to darkness treatment, while the transcript level of *ZjMPK6* was significantly increased at 4 days after darkness treatment, and then markedly decreased, but later increased again. The *ZjMKK* genes also exhibited distinct expression profiles in response to darkness treatment (Fig. 10). Three *ZjMKK* genes were down-regulated and only one gene was up-regulated as compared to the control in the darkness treatment. *ZjMKK2* and *ZjMKK3* displayed the similar expression patterns with *ZjMPK5* and *ZjMPK10*, respectively. Additionally, *ZjMKK4* expression profile was the same as *ZjMPK3*. Moreover, *ZjMKK1* and *ZjMPK6* exhibited the same expression patterns.

Evidence is accumulating that MAPK and MAPKK proteins are involved in responding to various biotic stresses. Thus, the expressions of these genes were examined in diseased plantlets infected by phytoplasma. As shown in Fig. 11, most of the *ZjMPK* genes were down-regulated under phytoplasma infection. Specially, the expression of *ZjMPK1*, *ZjMPK3*, *ZjMPK4* and *ZjMPK9* was decreased dramatically. The expression level of *ZjMKK1* and *ZjMKK3* in diseased plantlets was decreased significantly, but the expression of *ZjMKK2* was increased.

Overall, *ZjMPK*s and *ZjMKK*s display the different expression patterns, indicating that they may play different roles in response to various treatments.

## Discussion

So far, MAPK and MAPKK gene families have been identified and functionally characterized in many plants, such as 20 MAPKs and 10 MAPKKs in *Arabidopsis*, 15 MAPKs and 8 MAPKKs in rice, 26 MAPKs and 9 MAPKKs in apple, and so on [8, 10–13, 31]. However, no data set of MAPKs and MAPKKs is available for Chinese jujube. In this study, a comprehensive analysis of gene structure, phylogenetic relationship, chromosomal distribution and expression of *ZjMAPKs* and *ZjMAPKKs* were presented at genome level. A total of 10 MAPKs and 5 MAPKKs were first identified, and their proteins property, such as the isoelectric point and molecular weight, were also predicted. When comparing the structure of ZjMAPKs genes with other plants, the entire group A ZjMAPKs consisted of six exons in jujube, *Arabidopsis* and mulberry. Group B genes contained six exons in jujube, mulberry, tomato and poplar, while AtMPK5, AtMPK11 and AtMPK13 consisted of four exons in *Arabidopsis*. Group C ZjMAPKs consisted of only two exons with highly conserved sizes, which is similar to the other plants, including *Arabidopsis*, poplar and apple. Group D ZjMAPKs were complex in jujube, *Arabidopsis*, tobacco, poplar and apple, despite having the same number of exons. Chinese jujube and mulberry have the same total number genes which less than the other dicotyledons (Table 2), the main reason might be due to they do not experience the related whole genome duplication in the evolutionary process.

To date, more and more evidences suggested that the MAPK cascades are involved in biotic and abiotic stress response. The best characterized MAPKs are AtMPK3, AtMPK4 and AtMPK6 in *Arabidopsis* and their orthologs in other plant species, all of which are mainly activated by different stimulus including abiotic stresses, pathogens and oxidative stress [32]. It has been indicated that MAPK proteins classified in the same groups might serve similar functions in different species [10]. Based on sequence homology, MAPKs can be classified into four distinct subfamilies (group A, B, C and D) in plant. In *Arabidopsis thaliana*, AtMPKs of group A have been reported to be positive regulators of defense signaling [33], especially AtMPK3 and AtMPK6 have been described to mediate innate immunity [34]. In our present study, we found that Z*jMPK1* which belong to Group A was expressed at a markedly lower level in the diseased plantlets infected by phytoplasma (Fig. 11), and the phylogenetic tree (Fig. 5) indicates that AtMPK3, 6 and ZjMPK1 have close phylogenetic relationship. So we can infer the potential functions of ZjMPK1 from the known AtMPKs in *Arabidopsis*, that ZjMPK1 might play an important role in the process of plant-phytoplasma interaction.

Most plant MAPK cascade genes were response to abiotic and biotic stress, and a few genes have been proved to be involved in reproductive organ development. *Arabidopsis* AtMPK6 was involved in anther and embryo development [35]. Guan et al. also proved that AtMPK3/AtMPK6 in *Arabidopsis* were necessary to guide the direction of pollen tubes, but not in their growth [36]. Moreover, A MAPKKK gene has been successfully isolated from *Solanum chacoense* which involved in embryo sac and pollen development [37]. Another research of genome-wide analysis showed that one expansion class of MAPKKKs in *solanaceous* species played some specific roles in reproductive organ development [38]. Interestingly, in this study, *ZjMKK5* (homolog with MKK9 in *Arabidopsis* and other plants) was specific expressed in early stage of flower bud development, indicating its function is related to jujube flower development. However, previously there was no evidence that MKK9 homolog was involved in reproductive organ development. Among MAPK cascade, both MAPKKKs and MAPKs were reported to play roles in reproductive organ development, some MAPKKs must mediate in their process. We deduced that MKK9 just mediated this process. MKK9 directly phosphorylates and activates MPK6 [39–41], which was proved to involve in anther, pollen and embryo development [35, 36]. Hence how the *ZjMKK5* cascade regulates reproductive organ development in Chinese jujube need to elucidate by further studies.

## Conclusions

Ten MAPK and five MAPKK genes were identified from the genome database of Chinese jujube, and then a series of bioinformatics analysis were conducted. The expression analysis of ZjMAPK and ZjMAPKK genes using RT-PCR shown that majority of ZjMAPK and ZjMAPKK genes were expressed in all tested organs/tissues with considerable differences. Moreover, *ZjMKK5* was specific expressed in early development stage of jujube flower bud, indicating it plays some roles in reproductive organs development. The transcript expression results demonstrated that most ZjMAPK and ZjMAPKK genes were down-regulated in response to plant growth regulators, darkness treatment and phytoplasma infection.

Taken together, in this work we presented a comprehensive analysis of the *ZjMPK* and *ZjMKK* gene family. It would provide a better understanding of the complexity of MAPKs in plants and provide an important foundation for further functional study of MAPK and MAPKK family genes in Chinese jujube.

## Additional files

Additional file 1: Figure S1. Different stages of floral organ development. I: Small bud 1, diameter 0.5~1.0 mm; II: Small bud 2, diameter 1.0~1.5 mm; III: Middle bud, diameter 1.5~2.0 mm; IV: Big bud, diameter 2.0~2.5 mm; V: Yellow bud, diameter 2.5~3.0 mm; VI: Split bud, diameter 3.0~3.5 mm; VII: Full flower, diameter 3.5~5 mm. (PDF 125 kb)

Additional file 2: All sequence data used in this manuscript. (ZIP 36 kb)

Additional file 3: Table S1. The primer information of *ZjMAPKs* and *ZjMAPKKs*. (DOCX 17 kb)

Additional file 4: Figure S2. Relative expression of *ZjMKK5* gene analyzed by qRT-PCR in Chinese jujube floral organ at different development stages. *ZjACT* primers were used as the internal standard. The mean expression value was calculated from 3 independent replicates. The vertical bars indicate the standard deviation. Different lowercase and uppercase letters were defined as significant and highly significant difference, respectively. (PDF 93 kb)

## Abbreviations

6-BA: 6-benzyladenine
CDS: Coding sequence
GSDS: Gene Structure Display Server
IBA: Indole-3-butyric acid
JWB: Jujube witches’ broom
MAPK: Mitogen-activated protein kinases
MAPKK: Mitogen-activated protein kinases kinases
MS: Murashige and Skoog
NAA: α-Naphthalene acetic acid
NCBI: National Center for Biotechnology Information
NJ: Neighbor-joining
ORF: Open reading frame
qRT-PCR: Quantitative real-time PCR
RT-PCR: Reverse transcription PCR
