# Supplementary figures and images for: Genome-wide identification and analysis of MAPK and MAPKK gene family in Chinese jujube (Ziziphus jujuba Mill.)
Source: BMC Genomics. 2017 Nov 9;18:855. doi: 10.1186/s12864-017-4259-4 (PMC5680602; doi:10.1186/s12864-017-4259-4)

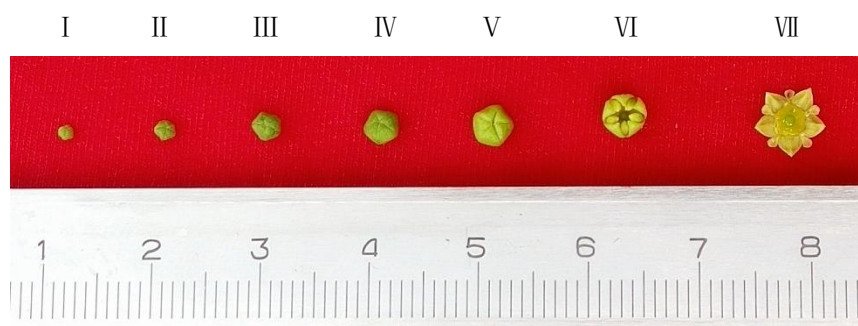

Supplement: Supplementary file 1 — Different stages of floral organ development. I: Small bud 1, diameter 0.5~1.0 mm; II: Small bud 2, diameter 1.0~1.5 mm; III: Middle bud, diameter 1.5~2.0 mm; IV: Big bud, diameter 2.0~2.5 mm; V: Yellow bud, diameter 2.5~3.0 mm; VI: Split bud, diameter 3.0~3.5 mm; VII: Full flower, diameter 3.5~5 mm. (PDF 125 kb) [file 12864_2017_4259_MOESM1_ESM.pdf]

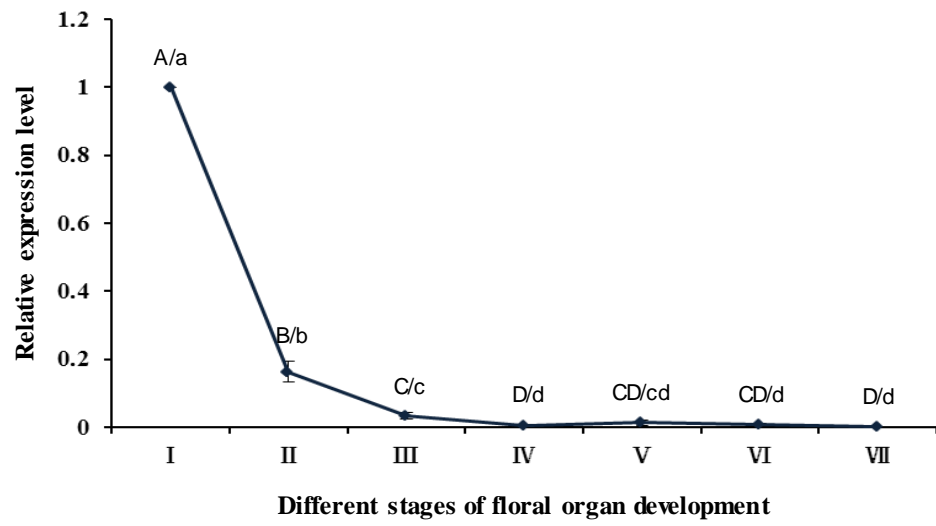

Supplement: Supplementary file 4 — Relative expression of ZjMKK5 gene analyzed by qRT-PCR in Chinese jujube floral organ at different development stages. ZjACT primers were used as the internal standard. The mean expression value was calculated from 3 independent replicates. The vertical bars indicate the standard deviation. Different lowercase and uppercase letters were defined as significant and highly significant difference, respectively. (PDF 93 kb) [file 12864_2017_4259_MOESM4_ESM.pdf]
