# Supplementary material for: Genome-wide identification and analysis of MAPK and MAPKK gene family in Chinese jujube (Ziziphus jujuba Mill.)
Source: BMC Genomics. 2017 Nov 9;18:855. doi: 10.1186/s12864-017-4259-4 (PMC5680602; doi:10.1186/s12864-017-4259-4)
Supplement: Supplementary file 3 — The primer information of ZjMAPKs and ZjMAPKKs. (DOCX 17 kb) [file 12864_2017_4259_MOESM3_ESM.docx]

**Table S1 The primer information of *ZjMAPKs* and *ZjMAPKKs***

|  | **Gene name** | **5’primer** | **3’primer** |
| --- | --- | --- | --- |
| MAPK | *ZjMPK1* | CCAGAGGGAGACATTTAACGA | GACAGTGCTCCTCCGACAAT |
|  | *ZjMPK2* | GGTTGCCATCAAGAAGATAG | ATTCCCTCCGTAAAGGTG |
|  | *ZjMPK3* | AACAGAATAGATGGCAAAAGGA | TCGCAACAACTGGTACAGAAAG |
|  | *ZjMPK4* | CTAGCCTTGGATTTCTTCG | TGTAATGCGTTTGTTGGGA |
|  | *ZjMPK5* | AAGCACTTCAACACCCTTACCT | TCTCCCCCAAATTCTCATCTAT |
|  | *ZjMPK6* | GGAGAGAAAGTTGCCATAAAGAA | ACAATATCAGGGTGCCGTAAAAG |
|  | *ZjMPK7* | GGCACTTGCTGATCCTTA | CTCCTTTGTTACCCTTCG |
|  | *ZjMPK8* | CTCAAACCATCCAAGGAGGA | TTCGTTGTTCAAGTGCCTCA |
|  | *ZjMPK9* | TTCTTGTCCTTCTCCTTCC | GGGTTTCAGACCCTTTACT |
|  | *ZjMPK10* | CGAACTCTGTGGGTCATTT | TTTCATTCCGTATCCTTGC |
| MAPKK | *ZjMKK1* | CTCGTGAATAGGGATGGAGT | AATGATTGTCTGTTGGCTGA |
|  | *ZjMKK2* | TAGTAAACCACAAAGGTGAGG | AGATTGCATATAAGGAAATCG |
|  | *ZjMKK3* | TGTTCTTTCGGCTATGTTTC | GCAGGCTTTATGTCTCTGTG |
|  | *ZjMKK4* | TTGAGGCTCTCCCTTCCACC | CCAAGAACCGCCAGTTTTTC |
|  | *ZjMKK5* | TGGGTCACTTCCCTCTGTT | CTGTGCTGCCGTCCATCTT |
|  | *ZjACT* | AGCCTTCCTGCCAACGAGT | TTGCTTCTCACCCTTGATGC |
